# Framework for a living systematic review and meta-analysis for the surgical treatment of bladder cancer: introducing EVIglance to urology

**DOI:** 10.1097/SP9.0000000000000008

**Published:** 2023-09-18

**Authors:** Victoria L.S. Wieland, Daniel Uysal, Pascal Probst, Maurizio Grilli, Caelán M. Haney, Marie A. Sidoti Abate, Luisa Egen, Manuel Neuberger, Giovanni E. Cacciamani, Maximilian C. Kriegmair, Maurice S. Michel, Karl-Friedrich Kowalewski

**Affiliations:** a Department of Urology and Urologic Surgery, University Medical Center Mannheim; b Library, Medical Faculty Mannheim, University of Heidelberg, Mannheim; c Urological Hospital Munich-Planegg, Planegg; d Department of Urology, University Hospital Leipzig, Leipzig, Germany; e Keck School of Medicine, Catherine and Joseph Aresty Department of Urology; f Artificial Intelligence (AI) Center at USC Urology, USC Institute of Urology, Los Angeles, California, USA; g Department of Surgery, Cantonal Hospital Thurgau, Frauenfeld, Switzerland

**Keywords:** bladder cancer, evidence-based medicine, living evidence, living systematic reviews, meta-analysis, urologic surgery

## Abstract

**Background:**

Knowledge of current and ongoing studies is critical for identifying research gaps and enabling evidence-based decisions for individualized treatment. However, the increasing number of scientific publications poses challenges for healthcare providers and patients in all medical fields to stay updated with the latest evidence. To overcome these barriers, we aim to develop a living systematic review and open-access online evidence map of surgical therapy for bladder cancer (BC), including meta-analyses.

**Methods:**

Following the guidelines provided in the Cochrane Handbook for Systematic Reviews of Interventions and the Preferred Reporting Items for Systematic Reviews and Meta-Analyses Statement, a systematic literature search on uro-oncological therapy in BC will be performed across various literature databases. Within the scope of a meta-analysis and living systematic review, relevant randomized controlled trials will be identified. Data extraction and quantitative analysis will be conducted, along with a critical appraisal of the quality and risk of bias of each study. The available research evidence will be entered into an open-access framework (www.evidencemap.surgery) and will also be accessible via the EVIglance app. Regular semi-automatic updates will enable the implementation of a real-living review concept and facilitate resource-efficient screening.

**Discussion:**

A regularly updated evidence map provides professionals and patients with an open-access knowledge base on the current state of research, allowing for decision-making based on recent evidence. It will help identify an oversupply of evidence, thus avoiding redundant work. Furthermore, by identifying research gaps, new hypotheses can be formulated more precisely, enabling planning, determination of sample size, and definition of endpoints for future trials.

## Introduction

Over the last decades, tremendous progress in perioperative care for bladder cancer (BC) surgery has been made. This included the development of enhanced recovery after surgery (ERAS) protocols, adoption of minimally invasive, in particular robotic-assisted surgery, and enhanced visualization methods during transurethral resection of bladder tumor (TURBT), for example, photodynamic diagnostics^1,2^. However, the safe implementation of new surgical technologies or techniques must be supported by well-conducted randomized controlled trials (RCTs) and should follow the IDEAL (Idea, Development, Exploration, Assessment, and Long-term follow-up) framework to allow an evidence-based approach^3,4^.

Due to the rapid development of new evidence, constantly keeping up with the latest research findings has become challenging for healthcare providers and patients^5,6^. Systematic reviews (SRs) and meta-analyses (MAs) can help summarize existing literature and provide a rapid rehearsal for healthcare providers concerning a research question. However, conducting MAs is a resource and time-consuming process that can cost up to $100 000 for one MA^7,8^ and takes an average of 67.3 weeks^9^. This results in a considerable delay from the initial literature search until completion, rendering some MAs outdated at the time of publication^10^. A further concern arises from the length of the extended update cycle for guidelines. An SR found that 40% of 25 manuals indicated a time frame of 2–3 years for updating^11^. In urology, the European Association of Urology (EAU) guidelines specify an annual update cycle as the time frame for guidelines, while the number of RCTs continues to increase. However, in recent years, there has been a notable rise not only in RCTs but also in SRs. A retrospective study observed an increase of more than 20 times in the number of SRs published over the past two decades, resulting in around 80 new SRs being published daily. Interestingly, the number of SRs has grown more significantly than the number of publications listed in PubMed^12^. This has led to considerable overlap of SRs, raising concerns about research redundancy and inefficiency. As a result, there have been calls for modifications to the current system of evidence synthesis. Conducting MAs has become an ‘epidemic’, with multiple MAs being completed on the same topic but with different results. For instance, Chapelle *et al*.^13^ provided an example of four original RCTs included in 20 published MAs published by 142 authors. Therefore, it is difficult for readers to accurately assess and correctly interpret the results of a MA.

As a potential solution, establishing open-access living SRs with network MAs could overcome the limitations of a standard SR by staying current with the latest evidence through regular updates^14^. Recently, Probst *et al*.^15,16^ developed an open-access platform for pancreatic surgery that exemplifies the concept of living SRs, providing an overview of existing evidence from RCTs in pancreatic surgery (www.evidencemap.surgery). Furthermore, the EVIglance app is freely available for Android and iOS (iPhone Operating System)-based mobile phones. Consequently, clinicians would have an alternative to guidelines that may not include the latest evidence but can offer their patients the most recent and scientifically validated therapy in a timely manner. Moreover, researchers can detect knowledge gaps and generate new research hypotheses.

We aim to introduce a living SR and MA in urology, focusing on surgical interventions for BC. Our approach involves thorough screening and critical appraisal of available data to ensure easy access to current evidence from RCTs. We will present this summarized evidence as an open-access evidence map on www.evidencemap.surgery. Additionally, we will provide access via the EVIglance app, enabling users to stay up-to-date with the latest findings. In this manner, we will establish an ever-evolving knowledge base that includes rigorous critical appraisal and statistical analysis of included studies. As a result, this approach will optimize resource allocation, and redundant studies on the same topic can be eliminated.

## Material and methods

The planned project is a living SR with MA that will facilitate patients’, clinicians’, and researchers’ access to a regularly updated version of the evidence on surgical treatment options for BC. The proposed study will follow the standard methodology following the Cochrane Handbook for Systematic Reviews of Interventions^17^ and the recommendations of the Study Center of the German Society of Surgery^18^ and will be reported according to the standard guidelines Preferred Reporting Items for Systematic Reviews and Meta-Analyses (PRISMA)^19^ (Supplemental Digital Content 2, http://links.lww.com/ISJP/A1 and Supplemental Digital Content 3, http://links.lww.com/ISJP/A2). The planned review follows the AMSTAR criteria as a guiding framework. To ensure transparency, we will conduct an AMSTAR-2 assessment after the completion of the review and make it available to the public^20^. Prospective registration at the international prospective registry for systematic reviews PROSPERO (CRD42021247145; https://www.crd.york.ac.uk/prospero/display_record.php?ID=CRD42021247145)^21^.

### Systematic literature search

Following Goossen *et al*., a comprehensive literature search will be conducted in several databases with surgical relevance^18^: Cochrane Central Register of Controlled Trials (CENTRAL) in Cochrane Database of Systematic Reviews, PubMed (via NCBI-Platform), and Web of Science. For identifying ongoing trials, searches will be performed in trial registries such as clinicaltrials.gov (https://www.clinicaltrials.gov/) and the World Health Organization International Clinical Trials Registry Platform (WHO ICTRP; www.who.int/icrtp/en/) search portal. The search will be conducted by a librarian (M.G.). No restrictions will be applied in terms of language or publication date. The systematic literature search will be conducted based on the following PICOS (Population, Intervention, Comparison, Outcomes and Study) criteria^22^ (see Table 1).

**Table 1:** Population, Intervention, Comparison, Outcomes, and Study (PICOS) design criteria as a framework for defining the research question and developing eligibility criteria.

*Population* Patients with BC requiring surgical treatment.
*Intervention* Any surgical intervention related to the treatment of bladder cancer (e.g. radical cystectomy).
*Comparison* Standard treatment or any other comparable intervention (in the case of multi-arm studies).
*Outcome* Primary endpoints include oncological results (overall survival, recurrence-free survival, quality of life). Secondary endpoints include all treatment-related outcomes or surrogate parameters (oncological and functional outcomes, perioperative parameters (e.g. complications, blood loss, transfusion rate, operating time, length of hospital stay). Due to the nature of the review, there is a possibility of adding endpoints during the work process.
*Study design* Randomized controlled trials.

BC, bladder cancer.

The complete search strategy is presented in the Supplementary Appendix (Supplemental Digital Content 1, http://links.lww.com/ISJP/A0). The number of hits for the first search for relevant articles is estimated to be ~10 000 articles. The number of each periodical update will be significantly lower. Results will be imported into the literature management program EndNote 20 (Clarivate Analytics, Philadelphia, US), and duplicates will be removed. Nonelectronic search results will be edited manually and added to EndNote.

### Study selection and data collection

The multistage screening, including all review steps, will be carried out by two independent reviewers according to the guidelines of the Cochrane Collaboration^17^. Disagreements in any of the steps will be resolved by consensus or consultation with a third reviewer. The titles and abstracts will be reviewed for relevance. Reaching an agreement between the two reviewers, the full text will be retrieved and screened for inclusion and exclusion criteria. If the inclusion criteria are not fulfilled, the full texts will be sorted out, and the respective reason for exclusion will be documented. The detailed selection process of the relevant literature will be summarized as a PRISMA flowchart.

### Exclusion criteria

Since RCTs represent the highest level of evidence, studies with other study designs will be excluded based on limitations.

### Multiple publications of the same study

Exact duplicates will be recognized as such by the literature management program EndNote 20 and will be removed. In the case of publication series, bias will be avoided by preventing multiple considerations of the results. However, different publications from the same study might be considered to capture all information.

### Data extraction

Data extraction will be performed using predetermined criteria. The extracted data will be entered into a data sheet created for this purpose (Microsoft Excel). The suitability of the data sheet will be tested in advance on five studies.

### Risk of bias

The quality assessment of every RCT will be conducted according to version 2 of the Cochrane risk-of-bias tool for randomized trials (RoB 2)^23^. The risk-of-bias tool includes the following five domains: bias due to the randomization process, bias due to deviations from the planned interventions, bias due to missing outcome data, bias in the measurement of outcomes, and bias in the selection of reported outcomes. These domains are classified as follows: ‘high risk of bias’, ‘low risk of bias’, or ‘some concern’^23^. An overall assessment of risk-of-bias will be provided and presented as traffic light plots.

### Quality of evidence

The GRADE (Grading of Recommendations Assessment, Development, and Evaluation) approach will be used to assess the certainty of the evidence for each outcome and to grade the strength of the recommendation. This approach critically evaluates the assessment of design, risk of bias, unexplained heterogeneity and inconsistency, indirectness of evidence, imprecision of results, and other factors such as publication bias. The certainty of the evidence can be rated as: ‘high’, ‘moderate’, ‘low’, and ‘very low’^24^. For transparent quality descriptions, each study’s quality characteristics will be compiled into a table.

### Statistical analysis

All retrieved data will be made freely available on the online platform. Therefore, forest plots will be used for visualization, and results will be reported descriptively and in a plain language summary. For continuous outcomes, the standardized mean difference (SMD) with its 95% CI will be reported as the effect measure per trial. The odds ratio (OR) with its 95% confidence interval (CI) will be used as the effect measure per trial for dichotomous outcomes. If possible, the OR and its standard error will be extracted directly, preferably from an adjusted model. Otherwise, if events and the number of patients per intervention group are available, the unadjusted OR will be obtained along with its standard error. The (adjusted) HR (hazard ratio) estimated in Cox regression models will be used when reported for survival outcomes. Alternatively, results from log-rank tests, Kaplan–Meier curves, or reported Kaplan–Meier estimates will be utilized. If neither Cox regression nor Kaplan–Meier estimators are reported, survival outcomes will be extracted as dichotomous outcomes.

The random effects model will be adopted due to possible clinical heterogeneity between studies. Results will be visualized using forest plots. Statistical in between-study heterogeneity will be expressed as *I* ^2^, indicating *I* ^2^<25% insignificant, *I* ^2^=25–50% low, *I* ^2^=50–75% moderate, and *I* ^2^>75% high^25^. A sensitivity analysis will be performed if a high level of heterogeneity is assumed. Values other than the mean and standard deviation (SD) will be transformed according to the methods described by Hozo *et al*.^26^ and Higgins *et al*.^17^.

Since it will be assumed that more than two interventions will be compared within a given endpoint and indication, state-of-the-art network MA will be performed. Depending on the scale of the endpoint, either linear (continuous or time-to-event data) or logistic (binary data) hierarchical random-effects models will be used. Pooled estimates of the mean mixed effect resulting from these analyses will be used along with their 95% prediction intervals^27^ to summarize the available evidence and account for between-study heterogeneity. The results will be presented graphically in forest plots along with the data from each study.

Pooling will only be performed if there exist more than three interventions within a research topic [e.g. robotic vs. laparoscopic vs. open radical cystectomy (RC)] and studies are similar enough (patient cohorts, tumor stage, prior treatment, indication, surgical technique). Funnel plots will be performed to detect possible publication bias. A statistical investigation of potential publication bias based on a test of funnel plot asymmetry will be conducted if there are at least 10 studies pooled for one outcome. A *P*-value ≤0.05 will be considered statistically significant.

R version 4.1.1 (R Foundation for Statistical Computing, Vienna, Austria) will be used for all statistical analyses. For generating diagrams, we will use the R package *meta* ^28^.

### Online platform

An open-access platform initially developed by Probst *et al*.^15^ (www.evidencemap.surgery) enables the concept of living SRs. It provides an overview of the existing evidence available from RCTs. The living and systematic evidence map allows a quantitative and visual representation of the current state of research, facilitating access to evidence for researchers, clinicians, and patients and their families. The knowledge base provides the available evidence as an intuitive mind map. In addition, forest plots, a summary of findings, and data extraction sheets will be provided. Ongoing studies will be included in the map and labeled as such. Existing gaps in the knowledge pool will be identified and highlighted through the creation of the evidence map. Publications from RCTs for BC surgery will be cited and linked to the full text.

**Figure 1:**
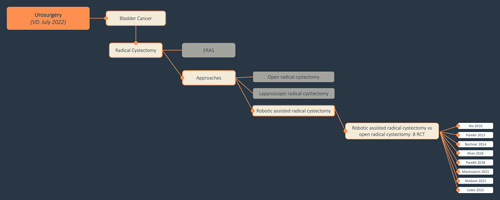
Exemplary representation of the possible structure of the evidence map. ERAS, enhanced recovery after surgery; RCT, randomized controlled trial.

The following figure (Fig. 1) provides an example of the structure of the planned evidence map for BC.

Social media platforms (e.g. Meta Platforms, Inc. or Twitter, Inc.) and the comment function included in the evidence map will enable an interactive exchange within the community. In this way, international researchers can announce new developments uncomplicatedly, but clinicians and patients benefit from this exchange to be alerted to the latest study results. The interactions of the individual users on the platform can also generate information about the need for further research topics.

In addition to the evidence map on pancreatic surgery created by Probst *et al*., the knowledge database is also available as a free app called EVIglance for Android and iPhone users.

### Updating

**Figure 2:**
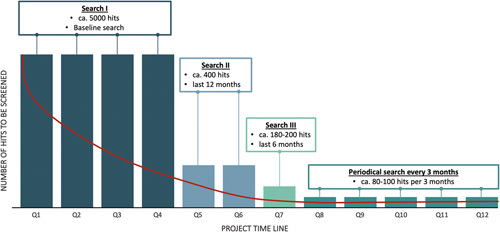
Long-term strategy and estimated hits for bladder cancer exemplary.

To obtain an approximate estimate of the workload required, a systematic literature search in Medline via PubMed was performed by a librarian (M.G.). BC was chosen as the case report, and only RCTs and meta-analyses were considered. The full search strategy yielded 5059 hits. The initial analyses will require considerably more time than the updates, which are expected to occur every 3 months in the later phases. Therefore, the results of the initial analysis will be provided within 12 months of project initiation, the results of the initial update within 6 months, and every 3 months thereafter. To test whether this approach is realistic, the above search will be performed a second time, but only for the last 3 months, generating 88 hits. This number seems reasonable to review at regular 3-month intervals (see Fig. 2). For recently acquired RCTs during the update, the review steps (literature search, abstract and full-text screening, data extraction) are run and added to the corresponding research topic. To identify the current status of the living SR, the date of the last and next planned update will be highlighted.

## Discussion

In an era of scientific research overload, it is challenging to keep up with the latest results^5,6^. While MAs summarize the results of multiple studies, they face considerable drawbacks in terms of required workload or redundant work. For instance, regarding BC, some comparisons of different interventions are already available, and redundant work exists.

Research questions of interest (with redundant and potentially outdated MAs):

1. Robotic versus laparoscopic versus open RC (Clement *et al*.^29^, Rai *et al*.^30^, Sathianathen *et al*.^31^);
2. ERAS versus standard of care (Cerantola *et al*.^32^, Giannarini *et al*.^33^, Tyson *et al*.^34^);
3. Monopolar versus bipolar TURBT (Sharma *et al*.^35^, Mao *et al*.^36^, Krajewski *et al*.^37^);
4. Photodynamic diagnosis versus white light TURBT (Kausch *et al.* ^38^, Maisch *et al.* ^39^, Veeratterapillay *et al.* ^40^).

Currently, evidence-based decision-making is based on the free processing of research results by guidelines. However, a disadvantage of guidelines is the limited annual updating with an additional turnover time of about 3–4 years. Consequently, the latest research results are only considered with a certain delay.

The relevance of this project additionally results from the market environment. In this context, the evidence-based resource ‘UpToDate’, a paid version of an interactive clinical decision support tool, should be mentioned. Especially in countries with lower financial resources, access to up-to-date, evidence-based medicine is limited due to the financial burden. This underlies the need for a database that is accessible to everyone free of charge.

Thus, new concepts are needed to tackle these limitations. The establishment of living SRs and network MAs with constant updating and innovative approaches to dissemination may help to overcome traditional barriers of high workload and availability of evidence.

An essential aspect of this work will be the usability of the evidence map for guideline development. Currently, most guidelines focus on aspects of medical oncology, while surgical oncology and especially surgical techniques, are partially neglected. However, the standardization of surgical care and dissemination of surgical evidence harbors the potential to enhance care greatly, as it is known that surgeons’ techniques can have a substantial impact on oncological outcomes. The continuous updates of the systematic review will enable guideline developers to keep up-to-date and react faster to published trials.

The proposed framework will provide proof of concept and introduce the idea of living SRs and evidence maps to urology. As a first scenario, all evidence for surgical treatment of BC will be analyzed and implemented on the online platform. This will enable clinicians to rely on up-to-date evidence and offer individualized therapy to their patients. At the same time, scientists can identify research gaps and drive future research topics through clear evidence maps.

The present project includes the features that are listed in Table 2.

**Table 2:** Purpose and features of the evidence map for surgical treatment of bladder cancer.

Purpose and features
1. Establishment of an open-access internet platform to serve as a knowledge base. Support clinical decision-making and provide evidence-based information to patients with BC and their caregivers. Identify research gaps and generate new research hypotheses. Tracking ongoing studies for affected patients interested in participating.
2. Critical evaluation of the methodological quality of all available evidence from RCTs.
3. Avoidance of redundant work.
4. Securing resources and changing the way systematic reviews and meta-analysis are conducted.
5. Facilitation of research collaboration.
6. Monitoring the evolution of research quality over time in the context of guideline implementation (e.g. CONSORT).
7. Periodically repeating and updating the literature searches, assessment, and statistical analyses to maintain the most current research findings available.
8. Community interaction on current research findings.
9. Motivating researchers to conduct evidence maps and living SRs.

BC, bladder cancer; CONSORT, Consolidated Standards of Reporting Trials; RCTs, randomized controlled trials; SRs, systematic reviews.

Furthermore, social platforms have become a vital resource in a world of digitalization. Thus, social media is also used in research and is used by urologists for scientific discussions and research exchanges^41^. Social media, such as Meta (Platforms) and Twitter, offer the opportunity to get information about current research results. In addition to knowledge acquisition, interaction with international researchers is also of critical importance. Another aspect of social media is the dissemination of scientific results^42^. By sharing and discussing new research results, new impulses for conducting future studies can be gained and influenced. Junior researchers can also get involved and find access to the research community more easily. The additional use of hashtags can cause the emergence of an online community to facilitate an interaction between medical professionals, researchers, and patients on a specific topic. In addition, a greater reach can be achieved through hashtags, as shown by the example of #SoMe4Surgery, which gained over 5000 followers within 2 and a half years^43^. To take advantage of social media, updates and live SRs will be posted on Facebook and Twitter and sent to researchers as well as journals.

### Limitations

Despite similarities, the proposed project will differ from traditional Cochrane reviews that conduct high-quality traditional MAs specific to a certain research question and provide a comprehensive scientific embedding of the research question. Given the tremendous effort required to perform Cochrane reviews, they cannot always provide an up-to-date living approach. Thus, from the provided platform, it will not be possible to draw the same conclusion as from Cochrane MAs. Still, it will allow rapid rehearsal of existing evidence, including methodological and statistical analyses, and link to original publications that can be analyzed in detail by the interested reader.

## Conclusion

In summary, in times of rapid increases in research findings and ever-changing evidence, a new approach to evidence-based medicine, especially for SRs and MAs is necessary. In the future, the proposed concept will be scaled up to different tumor entities (e.g. prostate cancer, kidney cancer), endourology, or conservative treatment. In addition, further development of surgical therapies for BC, especially given demographic changes, will be of great importance and must be assessed closely.

## Ethical approval

Not applicable.

## Consent

Not applicable.

## Sources of funding

Open-access publication charges were founded by Deutsche Forschungsgemeinschaft within the funding program ‘Open-Access Publikationskosten’ and by Heidelberg University.

## Author contribution

All authors had full access to all the data in the study and takes responsibility for the integrity of the data and the accuracy of the data analysis. V.L.S.W., K.-F.K., P.P., and C.M.H.: study concept and design; V.L.S.W., K.-F.K., D.U., M.G., and M.A.S.A.: acquisition of data; V.L.S.W., K.-F.K., D.U., C.M.H., L.E., M.N., and G.E.C.: analysis and interpretation of data; V.L.S.W. and K.-F.K.: drafting of the manuscript; all authors: critical revision of the manuscript for important intellectual content; V.L.S.W. and K.-F.K.: statistical analysis; V.L.S.W. and K.-F.K.: obtaining funding; V.L.S.W. and K.-F.K.: administrative, technical, or material support; M.S.M. and M.C.K.: supervision; none: other.

## Conflicts of interest disclosure

All authors declare that they have no conflicts of interest.

## Research registration unique identifying number (UIN)

1. Name of the registry: PROSPERO.
2. Unique identifying number or registration ID: CRD42021247145.
3. Hyperlink to your specific registration (must be publicly accessible and will be checked): https://www.crd.york.ac.uk/prospero/display_record.php?ID=CRD42021247145

## Guarantor

Karl-Friedrich Kowalewski and Victoria L.S. Wieland were involved in the conceptualization and initial drafting of the paper. All authors were involved in the methodology, reviewing, and editing of the manuscript.

## Data availability statement

We hereby acknowledge that all datasets generated and/or analyzed as part of this study are publicly available upon reasonable request.

## Provenance and peer review

Not commissioned, externally peer-reviewed.

## Supplementary Material

SUPPLEMENTARY MATERIAL
