# Supplementary material for: Framework for a living systematic review and meta-analysis for the surgical treatment of bladder cancer: introducing EVIglance to urology
Source: Int J Surg Protoc. 2023 Sep 18;27(2):9–15. doi: 10.1097/SP9.0000000000000008 (PMC10688537; doi:10.1097/SP9.0000000000000008)
Supplement: SUPPLEMENTARY MATERIAL [file sp9-27-09-s001.docx]

# **Supplementary material:**

Search Strategy:

**PubMed**

| **Trefferzahl** | **Datum** |
| --- | --- |
| 3864 | 27.11.2020 |

**P**

| "Urinary Tract Neoplas*"[tiab] OR  "Urinary Tract Cancer*"[tiab] OR  "Urinary Tract tumor*"[tiab] OR  "Urinary Tract tumour*"[tiab] OR  "Urinary Tract carcinoma*"[tiab] OR  "Urinary Tract adenocarcinoma*"[tiab] OR  "neoplasm of Urinary Tract"[tiab] OR  "Cancer of Urinary Tract"[tiab] OR  "tumor of Urinary Tract"[tiab] OR  "tumour of Urinary Tract"[tiab] OR  "neoplasm of the Urinary Tract"[tiab] OR  "Cancer of the Urinary Tract"[tiab] OR  "tumor of the Urinary Tract"[tiab] OR  "tumour of the Urinary Tract"[tiab] OR | 68338 |
| --- | --- |
| "urothelial Neoplas*"[tiab] OR  "urothelial Cancer*"[tiab] OR  "urothelial tumor*"[tiab] OR  "urothelial tumour*"[tiab] OR  "urothelial carcinoma*"[tiab] OR  "urothelial adenocarcinoma*"[tiab] OR |  |
| "Bladder Neoplas*"[tiab] OR  "Bladder Cancer*"[tiab] OR  "Bladder Tumor*"[tiab] OR  "Bladder Tumour*"[tiab] OR  "Bladder carcinoma*"[tiab] OR  "Bladder adenocarcinoma*"[tiab] OR  "neoplasm of Bladder"[tiab] OR  "Cancer of Bladder"[tiab] OR  "Tumor of Bladder"[tiab] OR  "Tumour of Bladder"[tiab] OR  "Carcinoma of Bladder"[tiab] OR  "adenocarcinoma of Bladder"[tiab] OR  "neoplasm of the Bladder"[tiab] OR  "Cancer of the Bladder"[tiab] OR  "Tumor of the Bladder"[tiab] OR  "Tumour of the Bladder"[tiab] OR  "Carcinoma of the Bladder"[tiab] OR  "adenocarcinoma of the Bladder"[tiab] OR  "neoplasm of Urinary Bladder"[tiab] OR  "Cancer of Urinary Bladder"[tiab] OR  "Tumor of Urinary Bladder"[tiab] OR  "Tumour of Urinary Bladder"[tiab] OR  "Carcinoma of Urinary Bladder"[tiab] OR  "adenocarcinoma of Urinary Bladder"[tiab] OR  "neoplasm of the Urinary Bladder"[tiab] OR  "Cancer of the Urinary Bladder"[tiab] OR  "Tumor of the Urinary Bladder"[tiab] OR  "Tumour of the Urinary Bladder"[tiab] OR  "Carcinoma of the Urinary Bladder"[tiab] OR  "adenocarcinoma of the Urinary Bladder"[tiab] OR |  |
| **"Carcinoma, Transitional Cell"[Mesh] OR**  "Transitional cell neoplas*"[tiab] OR  "Transitional cell cancer*"[tiab] OR  "Transitional cell tumor*"[tiab] OR  "Transitional cell tumour*"[tiab] OR  "Transitional cell carcinoma*"[tiab] OR  "Transitional cell adenocarcinoma*"[tiab] OR |  |
| "Urethral neoplas*"[tiab] OR  "Urethral tumor*"[tiab] OR  "Urethral tumour*"[tiab] OR  "Urethral cancer*"[tiab] OR  "Urethral carcinoma*"[tiab] OR  "Urethral Adenocarcinoma*"[tiab] OR  "Urethral squamous cell papilloma"[tiab] OR  "Adenocarcinoma of the urethra"[tiab] OR  "Adenocarcinoma of urethra"[tiab] OR  "Neoplasm of the urethra"[tiab] OR  "Neoplasm of urethra"[tiab] OR  "Cancer of the urethra"[tiab] OR  "Cancer of urethra"[tiab] OR  "Carcinoma of the urethra"[tiab] OR  "Carcinoma of urethra"[tiab] OR  "Tumor of the urethra"[tiab] OR  "Tumor of urethra"[tiab] OR  "Tumour of the urethra"[tiab] OR  "Tumour of urethra"[tiab] OR |  |
| "Ureteral neoplas*"[tiab] OR  "Ureteral cancer*"[tiab] OR  "Ureteral tumor*"[tiab] OR  "Ureteral tumour*"[tiab] OR  "Ureteral cell tumour*"[tiab] OR  "Ureteral carcinoma*"[tiab] OR  "Ureter cancer*"[tiab] OR  "Neoplasm of the ureter"[tiab] OR  "Neoplasm of ureter"[tiab] OR  "Cancer of the ureter"[tiab] OR  "Cancer of ureter"[tiab] OR  "Carcinoma of the ureter"[tiab] OR  "Carcinoma of ureter"[tiab] OR  "Tumor of the ureter"[tiab] OR  "Tumor of ureter"[tiab] OR  "Tumour of the ureter"[tiab] OR  "Tumour of ureter"[tiab] OR  "Adenocarcinoma of the ureter"[tiab] OR  "Adenocarcinoma of ureter"[tiab] OR |  |
| "Renal pelvis neoplas*"[tiab] OR  "Renal pelvis cancer*"[tiab] OR  "Renal pelvis tumor*"[tiab] OR  "Renal pelvis tumour*"[tiab] OR  "Renal pelvis carcinoma*"[tiab] OR  "Neoplasm of the renal pelvis"[tiab] OR  "Neoplasm of renal pelvis"[tiab] OR  "Cancer of the renal pelvis"[tiab] OR  "Cancer of renal pelvis"[tiab] OR  "Carcinoma of the renal pelvis"[tiab] OR  "Carcinoma of renal pelvis"[tiab] OR  "Tumor of the renal pelvis"[tiab] OR  "Tumor of renal pelvis"[tiab] OR  "Tumour of the renal pelvis"[tiab] OR  "Tumour of renal pelvis"[tiab] OR  "Adenocarcinoma of the renal pelvis"[tiab] OR  "Adenocarcinoma of renal pelvis"[tiab] |  |

**Filter RCT**

Nach Cochrane Handbuch

**Box 3.b Narrow mode:** **<https://training.cochrane.org/handbook/current/chapter-04-technical-supplement-searching-and-selecting-studies#_Ref19195069>**

| **clinical trials as topic [mesh: noexp] OR**  randomized controlled trial[pt] OR  controlled clinical trial[pt] OR  randomized[tiab] OR  placebo[tiab] OR  randomly[tiab] OR  trial[ti] | 1415089 |
| --- | --- |

**Cochrane Library**

| **Trefferzahl** | **Datum** |
| --- | --- |
| 4128 | 27.11.2020 |

**P**

| ("Urinary Tract Neoplas*"):ti,ab,kw OR  ("Urinary Tract Cancer*"):ti,ab,kw OR  ("Urinary Tract tumor*"):ti,ab,kw OR  ("Urinary Tract tumour*"):ti,ab,kw OR  ("Urinary Tract carcinoma*"):ti,ab,kw OR  ("Urinary Tract adenocarcinoma*"):ti,ab,kw OR  ("neoplasm of Urinary Tract"):ti,ab,kw OR  ("Cancer of Urinary Tract"):ti,ab,kw OR  ("tumor of Urinary Tract"):ti,ab,kw OR  ("tumour of Urinary Tract"):ti,ab,kw OR  ("neoplasm of the Urinary Tract"):ti,ab,kw OR  ("Cancer of the Urinary Tract"):ti,ab,kw OR  ("tumor of the Urinary Tract"):ti,ab,kw OR  ("tumour of the Urinary Tract"):ti,ab,kw OR  ("urothelial Neoplas*"):ti,ab,kw OR  ("urothelial Cancer*"):ti,ab,kw OR  ("urothelial tumor*"):ti,ab,kw OR  ("urothelial tumour*"):ti,ab,kw OR  ("urothelial carcinoma*"):ti,ab,kw OR  ("urothelial adenocarcinoma*"):ti,ab,kw OR  ("Bladder Neoplas*"):ti,ab,kw OR  ("Bladder Cancer*"):ti,ab,kw OR  ("Bladder Tumor*"):ti,ab,kw OR  ("Bladder Tumour*"):ti,ab,kw OR  ("Bladder carcinoma*"):ti,ab,kw OR  ("Bladder adenocarcinoma*"):ti,ab,kw OR  ("neoplasm of Bladder"):ti,ab,kw OR  ("Cancer of Bladder"):ti,ab,kw OR  ("Tumor of Bladder"):ti,ab,kw OR  ("Tumour of Bladder"):ti,ab,kw OR  ("Carcinoma of Bladder"):ti,ab,kw OR  ("adenocarcinoma of Bladder"):ti,ab,kw OR  ("neoplasm of the Bladder"):ti,ab,kw OR  ("Cancer of the Bladder"):ti,ab,kw OR  ("Tumor of the Bladder"):ti,ab,kw OR  ("Tumour of the Bladder"):ti,ab,kw OR  ("Carcinoma of the Bladder"):ti,ab,kw OR  ("adenocarcinoma of the Bladder"):ti,ab,kw OR  ("neoplasm of Urinary Bladder"):ti,ab,kw OR  ("Cancer of Urinary Bladder"):ti,ab,kw OR  ("Tumor of Urinary Bladder"):ti,ab,kw OR  ("Tumour of Urinary Bladder"):ti,ab,kw OR  ("Carcinoma of Urinary Bladder"):ti,ab,kw OR  ("adenocarcinoma of Urinary Bladder"):ti,ab,kw OR  ("neoplasm of the Urinary Bladder"):ti,ab,kw OR  ("Cancer of the Urinary Bladder"):ti,ab,kw OR  ("Tumor of the Urinary Bladder"):ti,ab,kw OR  ("Tumour of the Urinary Bladder"):ti,ab,kw OR  ("Carcinoma of the Urinary Bladder"):ti,ab,kw OR  ("adenocarcinoma of the Urinary Bladder"):ti,ab,kw OR  [mh "Carcinoma, Transitional Cell"] OR  ("Transitional cell neoplas*"):ti,ab,kw OR  ("Transitional cell cancer*"):ti,ab,kw OR  ("Transitional cell tumor*"):ti,ab,kw OR  ("Transitional cell tumour*"):ti,ab,kw OR  ("Transitional cell carcinoma*"):ti,ab,kw OR  ("Transitional cell adenocarcinoma*"):ti,ab,kw OR  ("Urethral neoplas*"):ti,ab,kw OR  ("Urethral tumor*"):ti,ab,kw OR  ("Urethral tumour*"):ti,ab,kw OR  ("Urethral cancer*"):ti,ab,kw OR  ("Urethral carcinoma*"):ti,ab,kw OR  ("Urethral Adenocarcinoma*"):ti,ab,kw OR  ("Urethral squamous cell papilloma"):ti,ab,kw OR  ("Adenocarcinoma of the urethra"):ti,ab,kw OR  ("Adenocarcinoma of urethra"):ti,ab,kw OR  ("Neoplasm of the urethra"):ti,ab,kw OR  ("Neoplasm of urethra"):ti,ab,kw OR  ("Cancer of the urethra"):ti,ab,kw OR  ("Cancer of urethra"):ti,ab,kw OR  ("Carcinoma of the urethra"):ti,ab,kw OR  ("Carcinoma of urethra"):ti,ab,kw OR  ("Tumor of the urethra"):ti,ab,kw OR  ("Tumor of urethra"):ti,ab,kw OR  ("Tumour of the urethra"):ti,ab,kw OR  ("Tumour of urethra"):ti,ab,kw OR  ("Ureteral neoplas*"):ti,ab,kw OR  ("Ureteral cancer*"):ti,ab,kw OR  ("Ureteral tumor*"):ti,ab,kw OR  ("Ureteral tumour*"):ti,ab,kw OR  ("Ureteral cell tumour*"):ti,ab,kw OR  ("Ureteral carcinoma*"):ti,ab,kw OR  ("Ureter cancer*"):ti,ab,kw OR  ("Neoplasm of the ureter"):ti,ab,kw OR  ("Neoplasm of ureter"):ti,ab,kw OR  ("Cancer of the ureter"):ti,ab,kw OR  ("Cancer of ureter"):ti,ab,kw OR  ("Carcinoma of the ureter"):ti,ab,kw OR  ("Carcinoma of ureter"):ti,ab,kw OR  ("Tumor of the ureter"):ti,ab,kw OR  ("Tumor of ureter"):ti,ab,kw OR  ("Tumour of the ureter"):ti,ab,kw OR  ("Tumour of ureter"):ti,ab,kw OR  ("Adenocarcinoma of the ureter"):ti,ab,kw OR  ("Adenocarcinoma of ureter"):ti,ab,kw OR  ("Renal pelvis neoplas*"):ti,ab,kw OR  ("Renal pelvis cancer*"):ti,ab,kw OR  ("Renal pelvis tumor*"):ti,ab,kw OR  ("Renal pelvis tumour*"):ti,ab,kw OR  ("Renal pelvis carcinoma*"):ti,ab,kw OR  ("Neoplasm of the renal pelvis"):ti,ab,kw OR  ("Neoplasm of renal pelvis"):ti,ab,kw OR  ("Cancer of the renal pelvis"):ti,ab,kw OR  ("Cancer of renal pelvis"):ti,ab,kw OR  ("Carcinoma of the renal pelvis"):ti,ab,kw OR  ("Carcinoma of renal pelvis"):ti,ab,kw OR  ("Tumor of the renal pelvis"):ti,ab,kw OR  ("Tumor of renal pelvis"):ti,ab,kw OR  ("Tumour of the renal pelvis"):ti,ab,kw OR  ("Tumour of renal pelvis"):ti,ab,kw OR  ("Adenocarcinoma of the renal pelvis"):ti,ab,kw OR  ("Adenocarcinoma of renal pelvis"):ti,ab,kw |  |
| --- | --- |

**CINAHL**

| **Trefferzahl** | **Datum** |
| --- | --- |
| 1296 | 27.11.2020 |

**P**

| "Urinary Tract Neoplas*" OR  "Urinary Tract Cancer*" OR  "Urinary Tract tumor*" OR  "Urinary Tract tumour*" OR  "Urinary Tract carcinoma*" OR  "Urinary Tract adenocarcinoma*" OR  "neoplasm of Urinary Tract" OR  "Cancer of Urinary Tract" OR  "tumor of Urinary Tract" OR  "tumour of Urinary Tract" OR  "neoplasm of the Urinary Tract" OR  "Cancer of the Urinary Tract" OR  "tumor of the Urinary Tract" OR  "tumour of the Urinary Tract" OR  "urothelial Neoplas*" OR  "urothelial Cancer*" OR  "urothelial tumor*" OR  "urothelial tumour*" OR  "urothelial carcinoma*" OR  "urothelial adenocarcinoma*" OR  "Bladder Neoplas*" OR  "Bladder Cancer*" OR  "Bladder Tumor*" OR  "Bladder Tumour*" OR  "Bladder carcinoma*" OR  "Bladder adenocarcinoma*" OR  "neoplasm of Bladder" OR  "Cancer of Bladder" OR  "Tumor of Bladder" OR  "Tumour of Bladder" OR  "Carcinoma of Bladder" OR  "adenocarcinoma of Bladder" OR  "neoplasm of the Bladder" OR  "Cancer of the Bladder" OR  "Tumor of the Bladder" OR  "Tumour of the Bladder" OR  "Carcinoma of the Bladder" OR  "adenocarcinoma of the Bladder" OR  "neoplasm of Urinary Bladder" OR  "Cancer of Urinary Bladder" OR  "Tumor of Urinary Bladder" OR  "Tumour of Urinary Bladder" OR  "Carcinoma of Urinary Bladder" OR  "adenocarcinoma of Urinary Bladder" OR  "neoplasm of the Urinary Bladder" OR  "Cancer of the Urinary Bladder" OR  "Tumor of the Urinary Bladder" OR  "Tumour of the Urinary Bladder" OR  "Carcinoma of the Urinary Bladder" OR  "adenocarcinoma of the Urinary Bladder" OR  "Transitional cell neoplas*" OR  "Transitional cell cancer*" OR  "Transitional cell tumor*" OR  "Transitional cell tumour*" OR  "Transitional cell carcinoma*" OR  "Transitional cell adenocarcinoma*" OR  "Urethral neoplas*" OR  "Urethral tumor*" OR  "Urethral tumour*" OR  "Urethral cancer*" OR  "Urethral carcinoma*" OR  "Urethral Adenocarcinoma*" OR  "Urethral squamous cell papilloma" OR  "Adenocarcinoma of the urethra" OR  "Adenocarcinoma of urethra" OR  "Neoplasm of the urethra" OR  "Neoplasm of urethra" OR  "Cancer of the urethra" OR  "Cancer of urethra" OR  "Carcinoma of the urethra" OR  "Carcinoma of urethra" OR  "Tumor of the urethra" OR  "Tumor of urethra" OR  "Tumour of the urethra" OR  "Tumour of urethra" OR  "Ureteral neoplas*" OR  "Ureteral cancer*" OR  "Ureteral tumor*" OR  "Ureteral tumour*" OR  "Ureteral cell tumour*" OR  "Ureteral carcinoma*" OR  "Ureter cancer*" OR  "Neoplasm of the ureter" OR  "Neoplasm of ureter" OR  "Cancer of the ureter" OR  "Cancer of ureter" OR  "Carcinoma of the ureter" OR  "Carcinoma of ureter" OR  "Tumor of the ureter" OR  "Tumor of ureter" OR  "Tumour of the ureter" OR  "Tumour of ureter" OR  "Adenocarcinoma of the ureter" OR  "Adenocarcinoma of ureter" OR  "Renal pelvis neoplas*" OR  "Renal pelvis cancer*" OR  "Renal pelvis tumor*" OR  "Renal pelvis tumour*" OR  "Renal pelvis carcinoma*" OR  "Neoplasm of the renal pelvis" OR  "Neoplasm of renal pelvis" OR  "Cancer of the renal pelvis" OR  "Cancer of renal pelvis" OR  "Carcinoma of the renal pelvis" OR  "Carcinoma of renal pelvis" OR  "Tumor of the renal pelvis" OR  "Tumor of renal pelvis" OR  "Tumour of the renal pelvis" OR  "Tumour of renal pelvis" OR  "Adenocarcinoma of the renal pelvis" OR  "Adenocarcinoma of renal pelvis" |  |
| --- | --- |

**Filter RCT**

Nach Cochrane-Handbuch: [**https://training.cochrane.org/handbook/current/chapter-04-technical-supplement-searching-and-selecting-studies#section-3-6-3**](https://training.cochrane.org/handbook/current/chapter-04-technical-supplement-searching-and-selecting-studies#section-3-6-3)

| MH randomized controlled trials OR  MH double‐blind studies OR  MH single‐blind studies OR  MH random assignment OR  MH pretest‐posttest design OR  MH cluster sample OR  TI (randomised OR randomized) OR  AB (random*) OR  TI (trial) OR  (MH (sample size) AND AB (assigned OR allocated OR control)) OR  MH (placebos) OR  PT (randomized controlled trial) OR  AB (control W5 group) OR  MH (crossover design) OR  MH (comparative studies) OR  AB (cluster W3 RCT) |  |
| --- | --- |

**Clinical Trial Gov**

[**http://www.clinicaltrials.gov/**](http://www.clinicaltrials.gov/)

| **Trefferzahl** | **Datum** |
| --- | --- |
| 3264 | 27.11.2020 |

**P**

| "Urinary Tract Neoplasm" OR  "Urinary Tract Cancer" OR  "Urinary Tract tumor" OR  "Urinary Tract tumour" OR  "Urinary Tract carcinoma" OR  "Urinary Tract adenocarcinoma" OR  "Neoplasmm of Urinary Tract" OR  "Cancer of Urinary Tract" OR  "tumor of Urinary Tract" OR  "tumour of Urinary Tract" OR  "Neoplasmm of the Urinary Tract" OR  "Cancer of the Urinary Tract" OR  "tumor of the Urinary Tract" OR  "tumour of the Urinary Tract" OR  "urothelial Neoplasm" OR  "urothelial Cancer" OR  "urothelial tumor" OR  "urothelial tumour" OR  "urothelial carcinoma" OR  "urothelial adenocarcinoma" OR  "Bladder Neoplasm" OR  "Bladder Cancer" OR  "Bladder Tumor" OR  "Bladder Tumour" OR  "Bladder carcinoma" OR  "Bladder adenocarcinoma" OR  "Neoplasmm of Bladder" OR  "Cancer of Bladder" OR  "Tumor of Bladder" OR  "Tumour of Bladder" OR  "Carcinoma of Bladder" OR  "adenocarcinoma of Bladder" OR  "Neoplasmm of the Bladder" OR  "Cancer of the Bladder" OR  "Tumor of the Bladder" OR  "Tumour of the Bladder" OR  "Carcinoma of the Bladder" OR  "adenocarcinoma of the Bladder" OR  "Neoplasmm of Urinary Bladder" OR  "Cancer of Urinary Bladder" OR  "Tumor of Urinary Bladder" OR  "Tumour of Urinary Bladder" OR  "Carcinoma of Urinary Bladder" OR  "adenocarcinoma of Urinary Bladder" OR  "Neoplasmm of the Urinary Bladder" OR  "Cancer of the Urinary Bladder" OR  "Tumor of the Urinary Bladder" OR  "Tumour of the Urinary Bladder" OR  "Carcinoma of the Urinary Bladder" OR  "adenocarcinoma of the Urinary Bladder" OR  "Transitional cell Neoplasm" OR  "Transitional cell cancer" OR  "Transitional cell tumor" OR  "Transitional cell tumour" OR  "Transitional cell carcinoma" OR  "Transitional cell adenocarcinoma" OR  "Urethral Neoplasm" OR  "Urethral tumor" OR  "Urethral tumour" OR  "Urethral cancer" OR  "Urethral carcinoma" OR  "Urethral Adenocarcinoma" OR  "Urethral squamous cell papilloma" OR  "Adenocarcinoma of the urethra" OR  "Adenocarcinoma of urethra" OR  "Neoplasmm of the urethra" OR  "Neoplasmm of urethra" OR  "Cancer of the urethra" OR  "Cancer of urethra" OR  "Carcinoma of the urethra" OR  "Carcinoma of urethra" OR  "Tumor of the urethra" OR  "Tumor of urethra" OR  "Tumour of the urethra" OR  "Tumour of urethra" OR  "Ureteral Neoplasm" OR  "Ureteral cancer" OR  "Ureteral tumor" OR  "Ureteral tumour" OR  "Ureteral cell tumour" OR  "Ureteral carcinoma" OR  "Ureter cancer" OR  "Neoplasmm of the ureter" OR  "Neoplasmm of ureter" OR  "Cancer of the ureter" OR  "Cancer of ureter" OR  "Carcinoma of the ureter" OR  "Carcinoma of ureter" OR  "Tumor of the ureter" OR  "Tumor of ureter" OR  "Tumour of the ureter" OR  "Tumour of ureter" OR  "Adenocarcinoma of the ureter" OR  "Adenocarcinoma of ureter" OR  "Renal pelvis Neoplasm" OR  "Renal pelvis cancer" OR  "Renal pelvis tumor" OR  "Renal pelvis tumour" OR  "Renal pelvis carcinoma" OR  "Neoplasmm of the renal pelvis" OR  "Neoplasmm of renal pelvis" OR  "Cancer of the renal pelvis" OR  "Cancer of renal pelvis" OR  "Carcinoma of the renal pelvis" OR  "Carcinoma of renal pelvis" OR  "Tumor of the renal pelvis" OR  "Tumor of renal pelvis" OR  "Tumour of the renal pelvis" OR  "Tumour of renal pelvis" OR  "Adenocarcinoma of the renal pelvis" OR  "Adenocarcinoma of renal pelvis" |  |
| --- | --- |
